# Increasing success and evolving barriers in the hepatitis C cascade of care during the direct acting antiviral era

**DOI:** 10.1371/journal.pone.0199174

**Published:** 2018-06-18

**Authors:** Autumn Zuckerman, Andrew Douglas, Sam Nwosu, Leena Choi, Cody Chastain

**Affiliations:** 1 Vanderbilt Specialty Pharmacy Services, Vanderbilt University Medical Center, Nashville, Tennessee, United States of America; 2 Belmont University, College of Pharmacy, Nashville, Tennessee, United States of America; 3 Department of Biostatistics, Vanderbilt University Medical Center, Nashville, Tennessee, United States of America; 4 Department of Medicine, Division of Infectious Diseases, Vanderbilt University Medical Center, Nashville, Tennessee, United States of America; Centers for Disease Control and Prevention, UNITED STATES

## Abstract

Barriers remain in the hepatitis C virus (HCV) cascade of care (CoC), limiting the overall impact of direct acting antivirals. This study examines movement between the stages of the HCV CoC and identifies reasons why patients and specific patient populations fail to advance through care in a real world population. We performed a single-center, ambispective cohort study of patients receiving care in an outpatient infectious diseases clinic between October 2015 and September 2016. Patients were followed from treatment referral through sustained virologic response. Univariate and multivariate analyses were performed to identify factors related to completion of each step of the CoC. Of 187 patients meeting inclusion criteria, 120 (64%) completed an evaluation for HCV treatment, 119 (64%) were prescribed treatment, 114 (61%) were approved for treatment, 113 (60%) initiated treatment, 107 (57%) completed treatment, and 100 (53%) achieved a sustained virologic response. In univariate and multivariate analyses, patients with Medicaid insurance were less likely to complete an evaluation and were less likely to be approved for treatment. Treatment completion and SVR rates are much improved from historical CoC reports. However, linkage to care following referral continues to be a formidable challenge for the HCV CoC in the DAA era. Ongoing efforts should focus on linkage to care to capitalize on DAA treatment advances and improving access for patients with Medicaid insurance.

## Introduction

The impact of direct-acting antivirals (DAA) on the hepatitis C virus (HCV) epidemic hinges on multiple steps from diagnosis, to referral, to evaluation, and finally treatment. With pharmaceutical advances in the field of HCV resulting in high cure rates once DAA treatment is completed, an increased emphasis is now being placed on care delivery and strategies to eliminate HCV.[1, 2] While new therapies provide ideal tools, both historical (pre-DAA HCV treatment) and modern barriers to care must be identified and addressed to eliminate HCV.

The majority of data evaluating the HCV Cascade of Care (CoC) was observed prior to DAA treatment becoming the standard of care. One of the most widely cited estimates of the HCV CoC in the United States (US) reported that as of July 2013, among 3.5 million people estimated to have chronic HCV, only 16% were prescribed treatment and 9% achieved sustained virologic response (SVR).[3] The impacts of DAA therapy on evaluation, access, and treatment have brought new advances and challenges within the CoC.[4–6]

This study examines the HCV CoC in one real-world clinic in the DAA era following referral to an HCV provider as well as identify barriers to successful CoC completion. Previous studies of the CoC in the DAA era have highlighted difficulty linking patients to care after diagnosis in specific populations, challenges accessing costly treatment, and high SVR rates in patients completing DAA therapy.[7–9] However, many studies have not identified specific case rationale for why individuals do not advance through the CoC. In this study, we sought to identify reasons why patients and specific patient populations did not advance through care. These findings provide meaningful insights to the HCV CoC in the DAA era and can drive appropriate allocation of resources to improve the care continuum.

## Methods

A single-center, ambispective cohort study of patients receiving care at the Vanderbilt University Medical Center (VUMC) Infectious Diseases (ID) Clinic was performed. Data was retrospectively collected from October 2015 to July 2016, and prospectively collected from August to September 2016. Patients are referred to the VUMC ID Clinic from local community providers, by self-referral, or through internal VUMC referrals, including patients seen in internal medicine clinics, screened through the emergency department, and those receiving human immunodeficiency virus (HIV) care at the Vanderbilt Comprehensive Care Clinic, Following a referral to the ID Clinic, an appointment is scheduled and a reminder letter is sent to the address listed in the electronic medical record (EMR) or provided by the referring provider.

The VUMC ID Clinic employs an integrated model of care for patients with HCV consisting of three physicians, one clinical pharmacist, one pharmacy technician, and one nurse. Within this program, the physician team provides clinical evaluation and assessment in preparation for DAA therapy. The pharmacist delivers comprehensive medication management, including an evaluation for regimen appropriateness, drug interaction screening and mitigation, patient education, and DAA monitoring. The pharmacist and pharmacy technician ensure ongoing access to DAA treatment from prescription to completion of prescribed therapy either through insurers or patient assistance programs (PAP).

Inclusion criteria were diagnosis of chronic HCV (ICD10 of B18.2) and a new referral to the clinic. Exclusion criteria included active hepatocellular carcinoma, cognitive impairment, life expectancy of less than 6 months, or patients who were in the midst of the CoC at the time of data analysis (September 2017). This study received approval and waiver of informed consent from the Vanderbilt University Institutional Review Board.

### Outcomes and cascade of care definitions

The primary endpoint evaluated was sustained virologic response (SVR) at least 12 weeks after treatment completion. Secondary endpoints included achievement of each individual stage in the CoC as well as time to treatment approval after DAA prescription. For the purposes of this study, the CoC represented the progression from referral to the VUMC ID clinic through HCV evaluation, prescription, initiation, and completion of treatment, and achievement of a SVR at least 12 weeks after completing treatment. Definitions of each step within the CoC are found in Table 1.

**Table 1:** Cascade of care definitions.

Cascade of Care	Required Element	Reason Required Element Not Met
Referred	Scheduled appointment in the Vanderbilt University Medical Center Infectious Diseases Clinic	Appointment cancelled or re-scheduled by the time of the originally scheduled appointment.
HCV Treatment Evaluation	Initial HCV evaluation by a prescribing provider	Patient did not attend appointment for evaluation of HCV infection by a prescribing provider
	Staging and baseline labs completed	Necessary work-up for a prescribing treatment including fibrosis staging and baseline labs were not completed
Prescribed Treatment	Completion of one of the following: —Benefits investigation with intent to prescribe therapy —Prescription generated for HCV treatment	Drug interactions preventing prescription
		Social barriers preventing treatment prescription
		Other (e.g. patient refusal, etc.)
Treatment Approved	Completion of one of the following: —Third Party approval —PAP approval through the drug manufacturer —Other means necessary to fiscally cover HCV treatment	Ineligible or not approved through insurance or drug manufacturer PAP
Treatment Initiated	Fulfillment of a prescription and administration of at least one tablet of the prescribed medication	Patient lost to follow-up
		Social barriers preventing treatment initiation
		Other medical care priorities
		Other (patient refusal, etc.)
Treatment Completed	Confirmed administration of the entire prescribed treatment course by patient self-report	Treatment discontinued
		Unknown/lost to follow-up
		Adverse effects prevented completion
Sustained Virologic Response	An undetectable HCV RNA at least 12 weeks after completing HCV treatment	Virologic failure
		Patient lost to follow-up

HCV: Hepatitis C Virus; PAP: Patient Assistance Program

When individual patients did not proceed through the CoC, data was collected from the EMR on the reason(s) for lack of advancement. A patient was considered lost to follow-up if ≥ 5 attempts to contact the patient were made by phone as well as a letter sent to the patient’s most recent address with no response over at least three months. If a patient missed an initial appointment in the ID Clinic, one outbound call was made by pharmacist and a voicemail was left if there was an option to do so.

### Data collection

Dates of clinic visits were obtained from the Epic scheduling system while all other outcomes were collected from the EMR. Patient characteristics assessed in the EMR prior to an evaluation included patient age, gender, ethnicity, insurer, and home zip code. When available in referring paperwork or the EMR, additional data points were collected including cirrhosis, active illicit substance use, HIV co-infection, insurance status, psychiatric history, and gender. Additional characteristics were confirmed at the time of evaluation, including: HCV genotype, HCV treatment history, fibrosis stage, HIV co-infection, history of and/or ongoing illicit substance or injection drug use (IDU), history of and/or ongoing alcohol abuse, and psychiatric disorder.

Medicaid recipients were identified as having Medicaid as a primary insurer for prescription coverage at the time of HCV prescription. Patients were considered co-infected with HIV if they were labelled with ICD10 code B20. Cirrhosis was defined as meeting any of the following criteria: anatomic ultrasound showing changes consistent with cirrhosis; ultrasound with acoustic radiation force impulse predicting F3-F4 or F4 fibrosis; FIB-4 score ≥3.25; Fibrosure of ≥0.72; or a liver biopsy with Metavir score F4. Diagnosed psychiatric disorder included patients labelled with an ICD10 including F01-F69 and F80-F99. Ongoing alcohol abuse was defined as >5 drinks on most days of the week as reported by the patient. Ongoing illicit substance or injection drug use (IDU) was defined as use within 3 months of evaluation as reported by the patient. The “baby boomer” age cohort was defined as any patient born between January 1, 1945 and December 31, 1965.

All patients were assessed for HCV treatment based on AASLD/IDSA Guidelines for the Treatment of Hepatitis C from the same group of providers working within the VUMC ID Clinic.

### Statistical analysis

Categorical variables were described using number of patients and percentage, while continuous variables were described using, mean, median, standard deviation, and interquartile range. Demographic characteristics selected a priori included gender, ethnicity, insurance type, HIV co-infection, cirrhosis, psychiatric disorder, ongoing illicit substance use, and baby boomer age cohort. Outcomes were binary variables for retained status (indicating movement from one step in the cascade to the next), medication approval status, as well as the time to medication approval (measured in days).

To further investigate associations between patient demographic characteristics and outcome variables, analyses were performed using logistic regression for binary outcomes, a cox proportional hazard model for time to event (i.e., medication approval) outcomes, and multiple linear regression model for continuous outcomes initially fit without adjusting for covariates. This was followed by multivariable models that controlled for gender, insurance type, HIV co-infection, cirrhosis, psychiatric disorder, and illicit substance use. For the analysis of time to medication approval using a multiple linear regression model, logarithmic transformation was performed to reduce skewedness. To avoid case-wise deletion of records with missing covariates, we employed a multiple imputation method with 10 imputation samples using predictive mean matching. All statistical analyses were performed using the programming language R version 3.3.0.

## Results

A total of 193 patients were referred to the VUMC ID Clinic for HCV infection that met inclusion criteria; six patients were actively progressing through the CoC and were excluded from this analysis. The majority were male (61%) and Caucasian (71%). Table 2 summarizes demographic information of those referred to clinic (n = 187) and those that completed HCV evaluation (n = 120). Most patients completing evaluation had genotype 1a infection (66%), were treatment naïve (90%), and did not have cirrhosis (77%).

**Table 2:** Baseline characteristics of patients referred and completing an evaluation.

Baseline Characteristics	N	Overall	Referred Evaluated
		N = 187	N = 120
**Age**	187	38.5, 51.0, 57.0 (47.5 ± 12.9)	34.0, 52.0, 57.0 (46.5 ± 14.4)
**Male**	187	115 (61.5%)	86 (71.7%)
**Race**	186		
White/Caucasian		132 (71.0%)	82 (68.3%)
African American		48 (25.8%)	34 (28.3%)
Other		6 (3.2%)	4 (3.3%)
**Insurance**	187		
Medicaid		60 (32.1%)	23 (19.2%)
Medicare		21 (11.2%)	18 (15.0%)
Medicare/Medicaid dual		19 (10.2%)	15 (12.5%)
Private		72 (38.5%)	55 (45.8%)
Other		15 (8.0%)	9 (7.5%)
**Cirrhosis**	125	29 (23.2%)	28 (23.3%)
**Genotype**	144		
1a		97 (67.4%)	79 (65.8%)
1b		19 (13.2%)	18 (15.0%)
2		11 (7.6%)	9 (7.5%)
3		15 (10.4%)	12 (10.0%)
4		1 (0.7%)	1 (0.8%)
6		1 (0.7%)	1 (0.8%)
**Treatment Naive**	141	128 (90.8%)	108 (90.0%)
**HIV^a^ positive**	149	59 (39.6%)	52 (43.3%)
**Psychiatric Disorder**^c^	149	61 (40.9%)	48 (40.0%)
**Active alcohol abuse^d^**	143	17 (11.9%)	10 (8.7%)
**History of alcohol abuse^d^**	147	60 (40.8%)	49 (41.2%)
**Active illicit substance use**^e^	145	27 (18.6%)	16 (13.3%)
**Ongoing IDU^b^**	144	7 (4.9%)	2 (1.7%)
**History of IDU^b^**	147	81 (55.1%)	62 (51.7%)

^a^HIV: Human Immunodeficiency Virus

^b^IDU: Injection drug use.

^c^Psychiatric Disorder defined as diagnosed ICD9/10 including F01-F69 and F80-F99

^d^>5 drinks on most days of the week

^e^Illicit Substance Use based on self-reported use.

N is the number of non-missing values.

For continuous variables a, b, c represent the lower quartile, a the median, and b the upper quartile c, with Mean and SD: $\bar{X}$ ± 1 SD.

Categorical variables are summarized with the n and percentage: n (%).

Tests used: Wilcoxon test for continuous variables and Pearson test for categorical variables.

Clinical baseline demographics were unable to confirmed consistently for patients referred not-evaluated, and therefore are not listed or analyzed.

**Fig 1:**
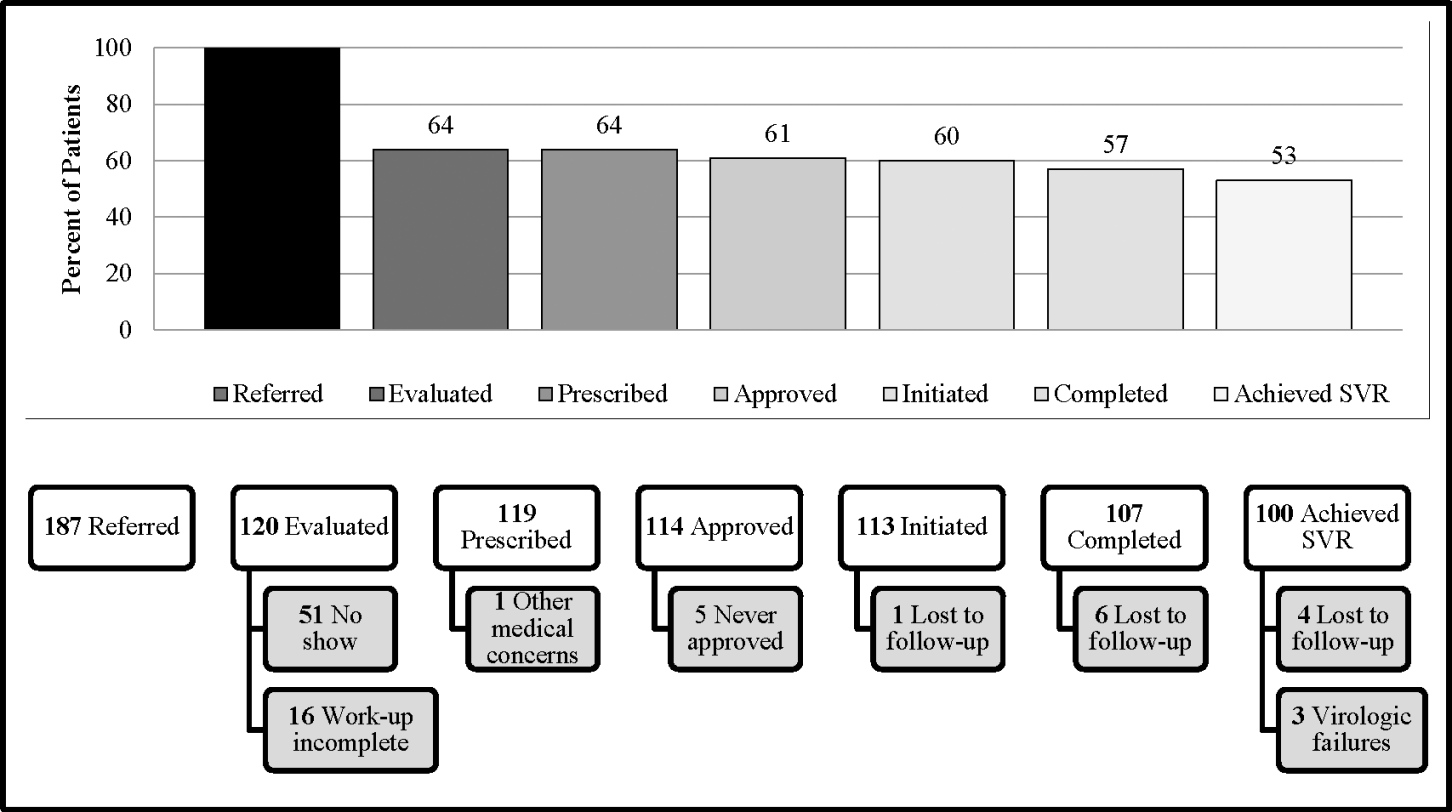
Cascade of care and reasons for lack of progression. Of 187 patients meeting inclusion criteria, 120 (64%) completed an evaluation for HCV treatment, 119 (64%) were prescribed treatment, 114 (61%) were approved for treatment, 113 (60%) initiated treatment, 107 (57%) completed treatment, and 100 (53%) achieved a SVR. The largest lack of progression was seen from a referral to an evaluation with 51 patients never attending a scheduled clinic appointment. After an evaluation was completed, the most common reason for lack of progression was losing a patient to follow-up, defined as ≥5 attempts to contact the patient were made by phone as well as a letter sent to the patient’s most recent address with no response over at least three months.

Of the 187 patients referred to the ID clinic, 120 patients (64%) completed evaluation for treatment, 119 (64%) were prescribed treatment, 114 (61%) were approved for treatment, 113 (60%) initiated treatment, 107 (57%) completed treatment, and 100 (53%) achieved an SVR. The largest drop between CoC stages occurred from referral to completing an evaluation (36%), including 51 (27%) patients who missed their scheduled appointment (i.e. were not linked to care) and 16 (9%) who never completed necessary work-up for treatment prescription and were lost to follow-up. One patient was not prescribed treatment due to other medical priorities. Of those that were prescribed treatment (n = 119), only five were never approved by insurance or through PAP (4%). Overall, 93% of all patients completing treatment achieved SVR; of those with available HCV RNA results at time of SVR evaluation (at least 12 weeks after treatment completion), 97% achieved SVR. The majority of patients that fell out of the CoC following an evaluation were lost to follow-up (9%). Reasons for lack of movement through the CoC at each stage are depicted in Fig 1.

In both univariate and multivariable logistic regression models, gender and insurance type were significantly associated with completing a clinic evaluation. After controlling for other factors, male patients were approximately 3 times more likely to complete an evaluation when compared to female patients (OR = 3.13, 95% CI = 1.50 to 6.55, *p* = 0.002). Additionally, after controlling for other factors, the odds of completing an evaluation decreased by 79% (OR = 0.21, 95% CI = 0.10 to 0.45, *p*<0.001) in patients with Medicaid (Table 3).

**Table 3:** Characteristics associated with evaluation completion.

Covariates	Evaluated N (%)^c^	Not Evaluated N (%)^c^	OR	95% C.I.	P-value
**Male** (ref = Female)	86 (71.7%)	38 (56.7%)	3.13	1.50–6.55	**0.002**
**Medicaid** (ref = Non-Medicaid)	23 (19.2%)	37 (55%)	0.21	0.10–0.45	**<0.001**
**HIV co-infection** (ref = No HIV co-infection)	52 (43.3%)	7 (24%)	1.46	0.57–3.72	0.426
**Psych Disorder** (ref = No Psych Disorder)^a^	48 (40.0%)	13 (44.8%)	0.91	0.26–3.18	0.881
**Illicit Substance Use** (ref = No Illicit Substance Use)^b^	16 (13.3%)	11 (44.0%)	0.41	0.14–1.21	0.106

HIV: Human Immunodeficiency Virus.

^a^Psychiatric Disorder defined as diagnosed ICD9/10 including F01-F69 and F80-F99.

^b^ Illicit Substance Use based on self-reported use or a positive value for illicit substances used on common drug screen.

^c^Percent of available data

Univariate analyses for investigating baseline patient characteristics and factors that may be associated with movement through the CoC found that patients with Medicaid were less likely to have treatment approved (*p* <0.001). No other baseline characteristics, including gender, HIV co-infection, cirrhosis, psychiatric disorder, and active illicit substance use were found to be significant when compared at any stage beyond evaluation within the CoC. All five patients never approved for treatment had Medicaid.

**Fig 2:**
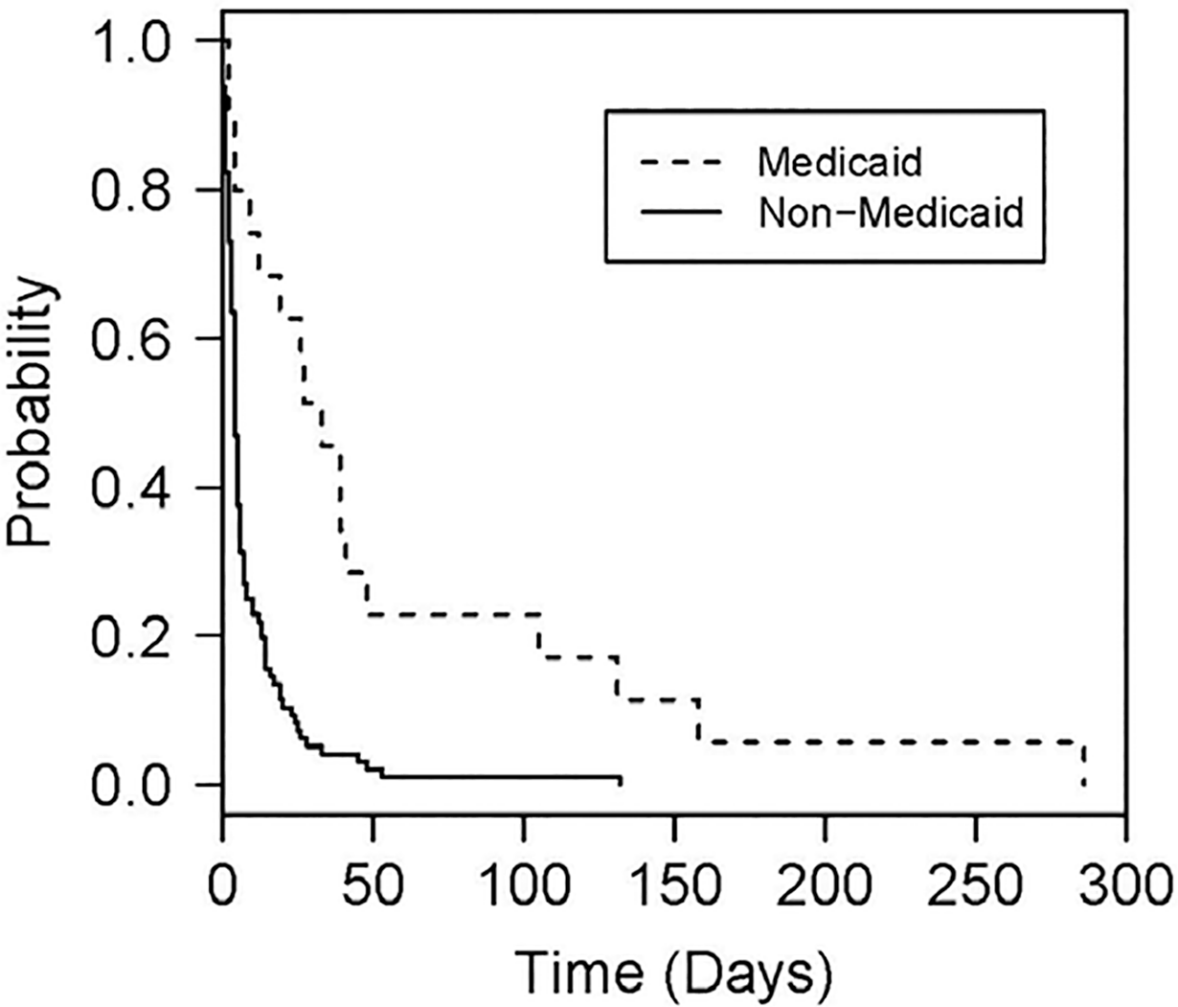
Time-to-approval analysis. Insurance type was a significant predictor of the rate in days to approval of direct acting antiviral therapy. The rate in days to approval decreased by 73% in patients with Medicaid compared with non-Medicaid (HR = 0.27, 95% CI = 0.15 to 0.48, p<0.001), reflecting a longer time to treatment approval in this population.

The median days to approval of treatment among patients who ultimately received treatment approval (n = 114) was five days (IQR 3–14). The time-to-event analysis indicated that insurance type and psychiatric disorder were important predictors associated with time to treatment approval after controlling for other factors. The rate in days to approval decreased by 73% in patients with Medicaid compared with non-Medicaid (HR = 0.27, 95% CI = 0.15 to 0.48, p<0.001), reflecting a longer time to treatment approval in this population (Fig 2). The median time to approval for patients with Medicaid was 30 days (SD 54 ± 73) compared to 4 days in non-Medicaid patients (SD 9±16). Conversely, the approval rate in days for patients with a psychiatric disorder increased relative to those without a psychiatric disorder, reflecting a shorter time to treatment approval in this population; however, this was not statistically significant (HR = 1.43, 95% CI = 0.95 to 2.16, p = 0.089). The multivariable linear regression analysis also showed that insurance type was associated with days to approval, indicating that patients with Medicaid had the geometric mean (GM) of 4.6 days longer time to approval when compared to non-Medicaid patients (GM = 4.6, 95% CI = 2.7 to 7.9, *p*<0.001).

## Discussion

This ambispective cohort study demonstrates that in our clinic, the majority of patients who completed an evaluation for HCV treatment also completed subsequent steps of the CoC and achieved an SVR. Despite modern challenges for HCV treatment including high pharmaceutical cost and a large population of patients with ongoing illicit substance use, this study demonstrated real-world efficacy of coordinated treatment programs.

### Linkage to care

As seen in historical and modern HCV CoC studies, linkage to care after referral was a significant barrier to CoC completion.[7, 8, 10, 11] Overall, 36% of patients with a scheduled appointment did not complete medical evaluation. Patients with Medicaid were 79% less likely to complete an evaluation as those without Medicaid, even when controlling for other factors. Previous studies have highlighted the disparity in this population obtaining access to treatment; however, our study demonstrates that this population may have difficulties accessing and completing other elements of medical care related to HCV.[6, 8, 9] Women were three times less likely to complete an evaluation in our cohort. While the reason for this gender disparity is likely complex, efforts should be focused on engaging women at the time of screening and referral, particularly in light of recent increased rates of HCV diagnosis in women of childbearing age and the subsequent risk of vertical transmission.[12]

Strategies to improve linkage to care should be further implemented and evaluated in the DAA era. These include engaging mental health and social work as well as coordinating substance abuse services for certain patients.[13] A number of interventions to improve linkage and access employed by the US Department of Veterans Affairs are translatable to those outside a single payer model and should be explored, including utilizing telemedicine and electronic technologies to reach rural and underserved areas, utilizing nonphysician advanced practice providers, and establishing services that address substance abuse.[14] As HCV treatment becomes more streamlined for most patients, shifting HCV treatment from specialty clinics to the primary care setting is an opportunity to improve effective linkage and completion of medical evaluation.[14, 15]

### Treatment prescription and initiation

Our results diverge with historical CoC models once patients completed evaluation, showing a high rate of retention in care with 89% of patients completing DAA treatment. Minimal loss between an evaluation and prescription of treatment occurred. Access through prior authorization (PA) approval to these costly medications has been identified as a barrier to DAA initiation.[6, 8, 9] We found a lower non-start rate than those previously reported in larger cohorts, with only 5% of our patients who were prescribed treatment not subsequently initiated.[5, 7, 16–18] Despite a large proportion of the evaluated cohort having Medicaid (32%), only five patients of 60 were not ultimately approved for treatment. Conversely, the TRIO Network recently reported a non-start rate of 48% in this population.[19] While Medicaid restrictions vary by state, Tennessee Medicaid restricts HCV treatment during the study period to patients with moderate to advanced fibrosis (F2 and above) with proof of alcohol and drug rehabilitation for any previous abuse as well as 6 months of sobriety.[20] The discrepancy in access to treatment by payer has been highlighted in multiple analyses and is perpetuated in this real world cohort.[5, 6, 18, 19, 21]

The median time to approval for all patients prescribed treatment was five days (IQR 3–14); lower than in several previously reported cohorts.[4, 5, 8, 19, 21] Patients without Medicaid (including those with no insurance) had a median time to approval of 4 days (IQR 2–8.5), while those with Medicaid who were approved had a median time of 30 days (IQR 10–46). Our results substantiate individual findings that for patients with Medicaid, delays in approval and initiation were common even if treatment is ultimately approved.[4, 22] Future studies should evaluate the clinical impact of treatment delays caused by difficulty accessing DAAs. Though not directly evaluated in this study, we believe our improved non-start rate and time to treatment approval are likely a result of the integrated model of our clinic with dedicated pharmacy services experienced in navigating the requirements to access therapy.

### Treatment completion

Few patients were lost to follow-up between treatment initiation and confirmed completion (n = 6), and no patients stopped treatment due to adverse effects or inability to afford treatment. This step within the CoC is clearly distinguishable from historical CoC data, where completion rates were lower due to the adverse effects of interferon and ribavirin.[23] Upon univariate analyses, no differences in treatment completion rates were seen among baseline characteristics studied. When medication approval was obtained, Medicaid patients and those with ongoing illicit substance use had similar rates of treatment and CoC completion. This finding further supports the AASLD/IDSA Guidelines’ recommendation to consider treatment in patients with ongoing illicit substance use.[24]

Within this study population, 95% of patients who initiated DAA therapy completed the full prescribed course. Provider fear of non-adherence to treatment has been highlighted as a reason for not prescribing DAA treatment.[25, 26] In the modern CoC, this concern is likely driven primarily by social factors including unstable housing, mental illness, or active alcohol and substance abuse rather than severe side effects from the medication.[14, 25, 27] Given the high prescription, initiation, and completion rates seen in the population in our study, concern regarding non-adherence may not be warranted in all settings. To facilitate initiation and completion of HCV treatment, providers should place an emphasis on engaging this population with social workers, case managers, and addiction counselors.[13, 14] Within our clinic, the pharmacist played an integral role in identifying and mitigating adverse effects that may have led to treatment discontinuation and enabled patients with counseling and adherence tools to ensure treatment completion. The impact of shorter treatment durations is yet to be elucidated but is likely only to improve completion rates.

### Sustained virologic response

Achievement of an SVR after treatment completion has clearly improved in the modern CoC with DAA treatment in comparison to historical standards.[28, 29] As expected, SVR rates for those that completed treatment were high (93%), with only 3 virologic failures among those with available laboratory data. Four patients were lost to follow-up after confirming treatment completion. A high intention-to-treat SVR rate was also seen in this real-world cohort, with 88% of patients who started treatment obtaining a confirmed SVR. None of the patients achieving an SVR in our evaluation were re-infected within the timeframe of the study. However, as more patients are treated and cured of HCV infection, future CoC studies should include movement beyond SVR eradication to include the frequency of patients who are re-infected.

### Limitations

All subjects were adults followed at a single outpatient ID clinic. A large percentage of patients were co-infected with HIV and few were actively using illicit drugs, particularly injection drugs. Therefore, the generalizability of the population is limited. As Medicaid restrictions differ by state, findings regarding delay in and access to treatment in Medicaid patients in our cohort may not reflect those of other Medicaid programs.[20] This study does not take into account the steps in the CoC prior to a referral to the clinic, including screening and diagnosis. This study was not blinded. Due to the limited sample size, the number of variables analyzed by multivariate analysis was limited. Finally, some factors that may have impacted an evaluation completion were not available, limiting a full analysis of these factors on evaluation completion rates.

## Conclusions

With DAA therapy as the new standard of care, the HCV CoC has transformed, still plagued by challenges in linkage to care yet substantially improved with regards to treatment outcomes. Interventions to emphasize screening, linkage to care, and access to treatment may address some of these challenges. Though DAA agents remain expensive for all groups, efforts to enhance and improve access across payer groups should be pursued. Integration of pharmacy services demonstrated high rates of medication access compared to previous studies, even in those with Medicaid. With new medications and modern tools, HCV treatment can be well-tolerated, effective, and result in high rates of completion.

## Supporting information

S1 Fig Optimizing the hepatitis C cascade of care in the direct-acting antiviral era. This data was presented as a poster at IDWeek 2017.
(PDF)
