# Supplementary material for: Increasing success and evolving barriers in the hepatitis C cascade of care during the direct acting antiviral era
Source: PLoS One. 2018 Jun 18;13(6):e0199174. doi: 10.1371/journal.pone.0199174 (PMC6005558; doi:10.1371/journal.pone.0199174)
Supplement: S1 Fig — This data was presented as a poster at IDWeek 2017. (PDF) [file pone.0199174.s001.pdf]

Optimizing the Hepatitis C Cascade of Care in the Direct-Acting Antiviral Era

Autumn D. Zuckerman, PharmD, BCPS, AAHIVP[1]; Andrew Douglas, MPH[2]; Karen Farris, PhD[3]; Cody A. Chastain, MD[4]

[1]Vanderbilt Specialty Pharmacy, [2]Belmont University School of Pharmacy, [3]University of Michigan, Department of Clinical Pharmacy, [4]Division of Infectious Diseases, Department of Medicine, Vanderbilt University Medical Center

BACKGROUND

- Despite improved treatments for Hepatitis C Virus (HCV) infection, barriers remain in the HCV cascade of care (CoC), limiting the overall impact of direct acting antivirals.
- The Vanderbilt Infectious Diseases (ID) clinic provides multidisciplinary care involving a physician, clinical pharmacist, and nurse for patients with HCV infection.
- The objective of this study was to identify factors associated with movement through the HCV CoC after referral to a multidisciplinary ID clinic to sustained virologic response (SVR), including both general and historically difficult to treat populations.

Clinical Pharmacist Responsibilities

Access	Education	Monitoring
- Obtaining medication access through insurers<br>- Procuring medication for uninsured<br>- Ensuring cost-effectiveness for patients<br>- Mitigating access barriers while on treatment	Prescribers:<br>- Treatment options to guide therapy decisions<br>Patients:<br>- Thorough medication overview and monitoring plan<br>- Creating adherence action plan<br>- Developing a follow-up plan	- Appropriate and timely pretreatment work-up<br>- Adherence, safety and efficacy monitoring by phone and in clinic<br>- Lab monitoring and dose adjustment when applicable<br>- Ensuring appointment adherence

METHODS

- Single-center, retrospective cohort study of patients receiving care at the VUMC ID Clinic between July 2015 and September 2016.
- Inclusion criteria: diagnosis of chronic HCV with an appointment in the VUMC ID clinic.
- Exclusion criteria: active carcinoma, cognitively impaired, life expectancy of ≤6 months.
- The following baseline characteristics were evaluated using a univariate analysis: HIV coinfection, patients with cirrhosis, people who use illicit substances (PWUIS), diagnosed psychiatric disorder, "Baby Boomer" (born between 1945-1965)

RESULTS

Baseline Characteristics	Referred (N = 187)	Evaluated (N=120)
**Age (mean ±SD)**	48±13	47±14
Baby Boomer	79 (42%)	69 (58%)
**Gender: p=0.0001†**		
Male	115 (62%)	86 (72%)
Female	72 (39%)	34 (28%)
**Ethnicity: p=0.011‡**		
White	132 (71%)	82 (68%)
African American	48 (26%)	34 (28%)
Other	3 (2%)	4 (3%)
**Insurance Type: p=<0.0001†**		
Medicare	21 (11%)	18 (15%)
Medicare/Medicaid	19 (10%)	15 (13%)
Medicaid	60 (32%)	23 (19%)
Private	72 (39%)	55 (46%)
Other	15 (8%)	9 (8%)

Differences between groups was assessed using Chi-Square† or Fisher's Exact‡

Baseline Characteristics of Patients Evaluated (N = 120)	
Genotype 1a	79 (66%)
Treatment naïve	108 (91%)
Cirrhosis	28 (24%)
HIV coinfection	51 (43%)
History of IVDU	62 (52%)
Ongoing IVDU*	2 (2%)
Ongoing alcohol use∫	10 (9%)
Ongoing illicit substance use*	15 (13%)
Psychiatric disorder	47 (40%)

*Denotes use within 3 months of evaluation
∫Denotes >5 drinks on most days of the week
IVDU= Intravenous drug use

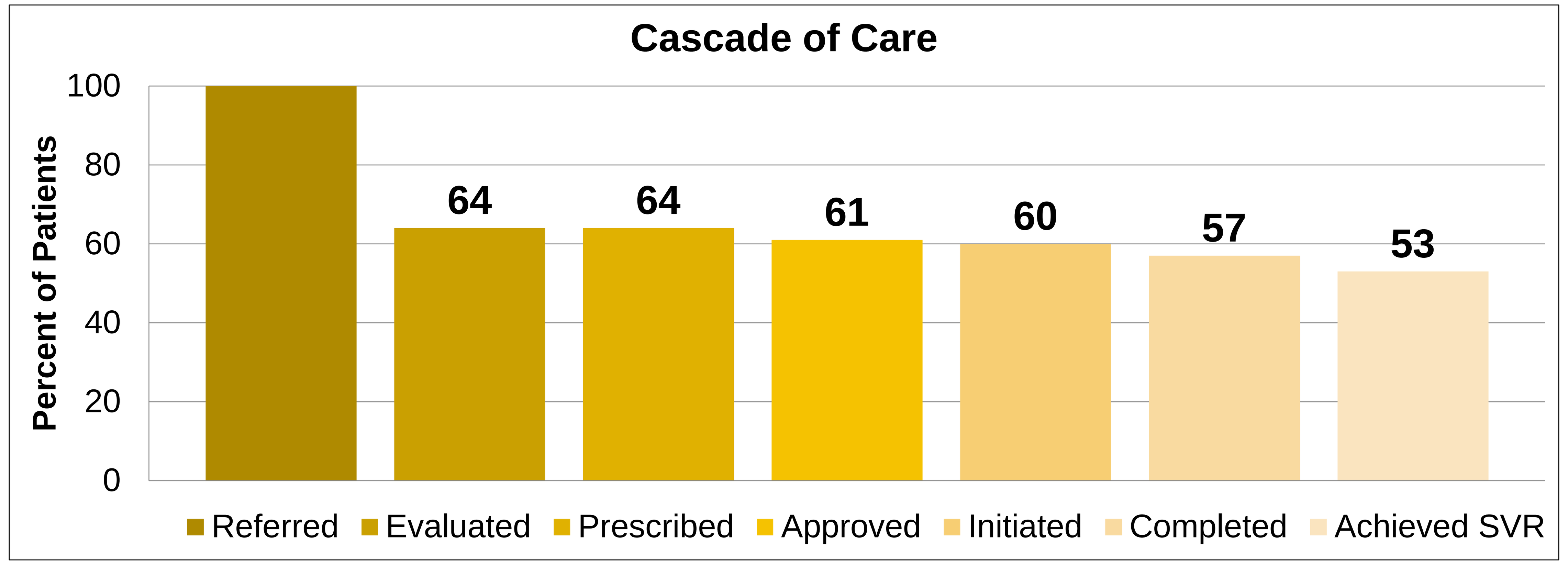

Movement Through the Cascade of Care

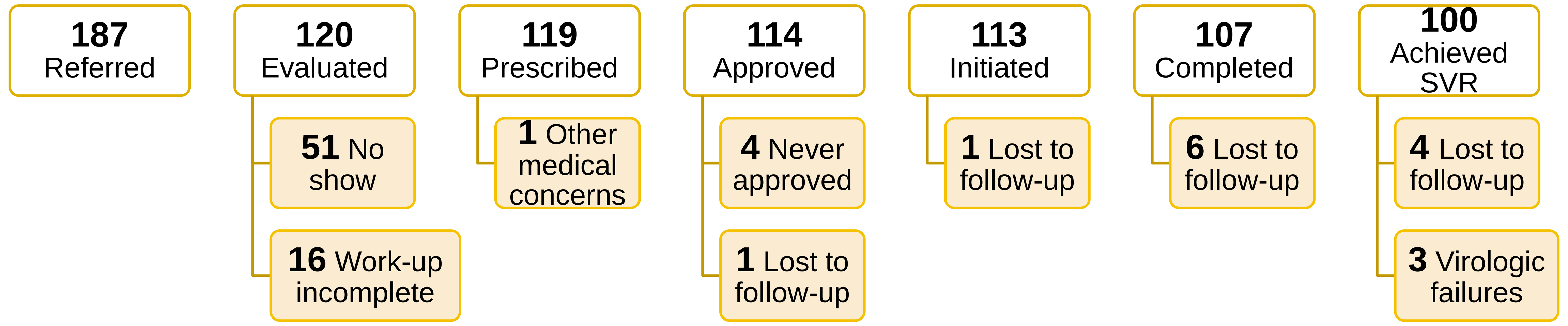

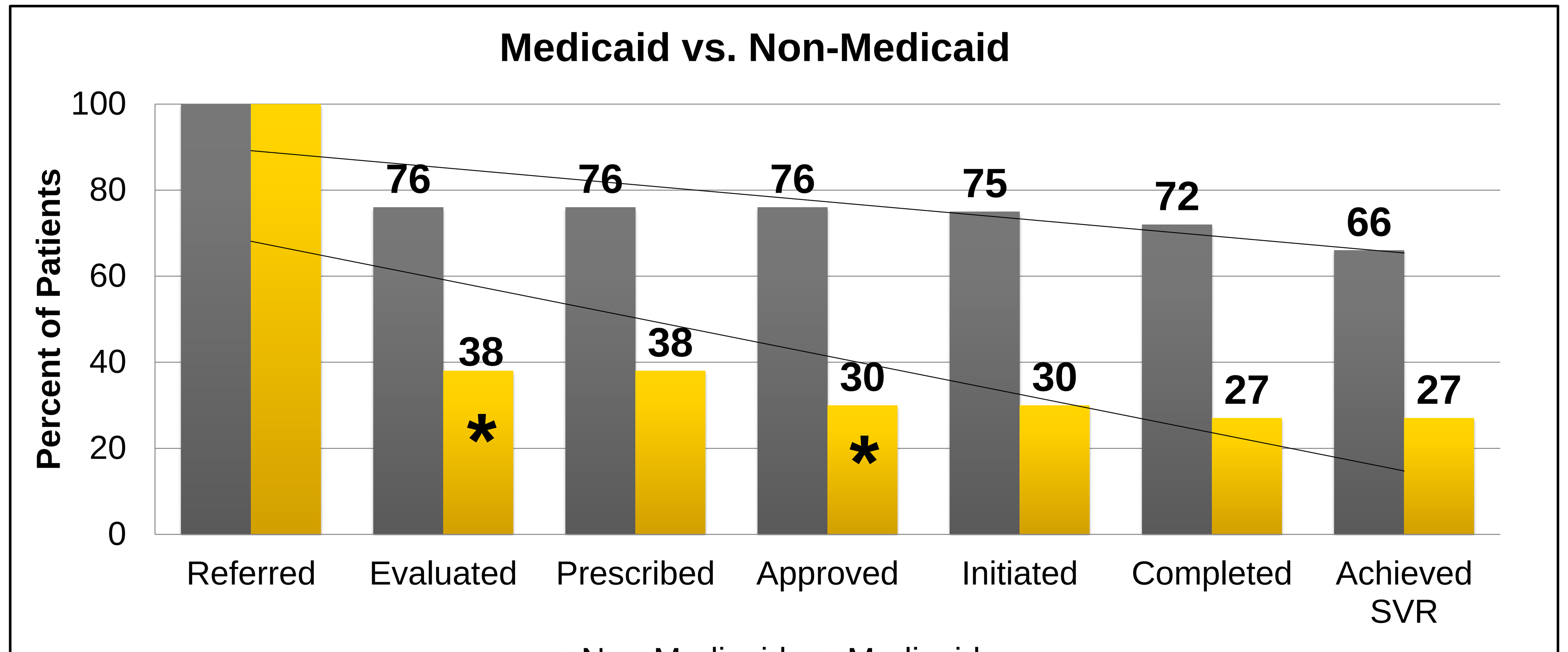

*Denotes statistical significance
Fisher's exact test was used to calculate p-values

RESULTS

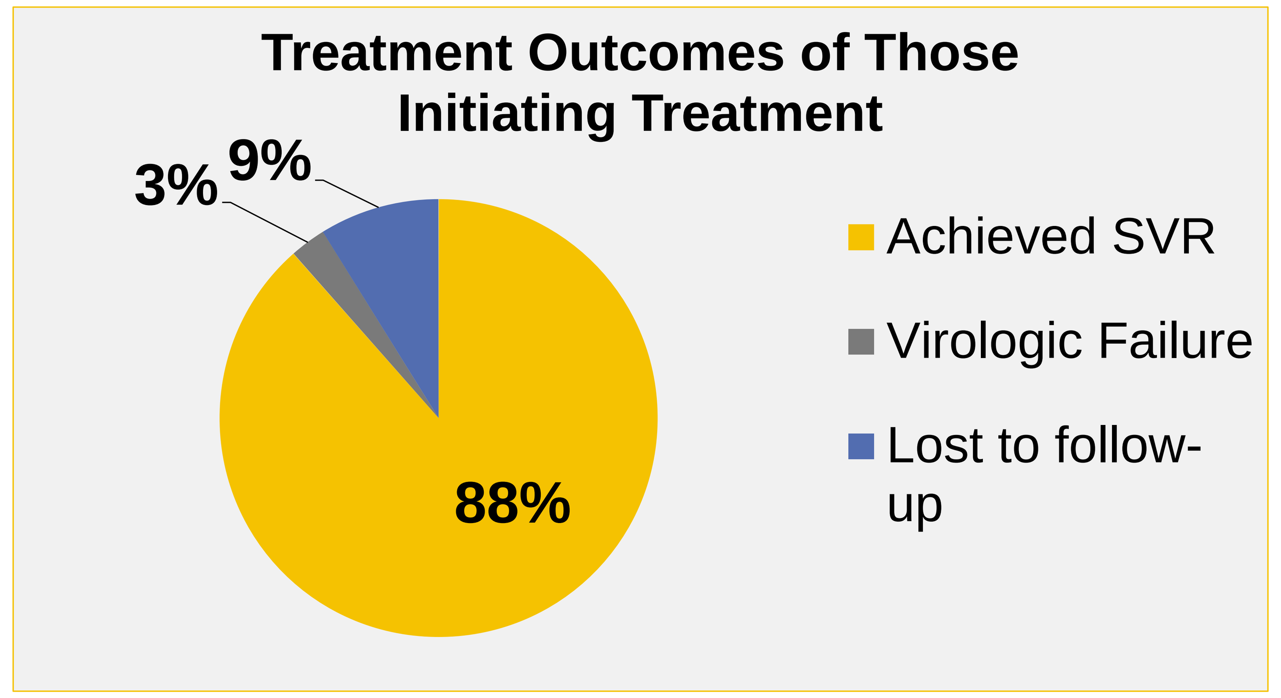

- SVR rate of 93.4% in patients who initiated and completed treatment, with only 3 virologic failures
- Of baseline characteristics compared, only having Medicaid insurance was associated with a lower rate of treatment approval.

DISCUSSION AND CONCLUSIONS

Recent real world reports of over 15,000 patients with HCV found that 37% of patients prescribed HCV treatment in 2016 did not actually initiate treatment.[2]

Conversely, within the ID clinic, 97% of patients prescribed treatment were initiated on treatment.

- Compared to previous reports of the HCV CoC, the ID clinic showed high rates of retention in care, treatment initiation, and SVR.
- Presence of baseline characteristics that have historically been identified as harder to treat were not significant predictors of lack of movement through the CoC.
- While not specifically evaluated by this study, we hypothesize that the high retention and completion rates observed was due to the integrated model of care delivery.

REFERENCES:

1. Yehia BR, Schranz AJ, Umscheid CA, Lo Re V, 3rd. The treatment cascade for chronic hepatitis C virus infection in the United States: a systematic review and meta-analysis. PLoS One. 2014;9(7):e101554. doi:10.1371/journal.pone.0101554.
2. Clough B, Afdhal N, Milligan S, Slotnik J. TRIO-Health Real-World Evidence: Hepatitis C Treatment Demand & Non-Starts. March 8th, 2107. http://www.natap.org/2017/HCV/TrioHealthTroutGroupHepC.PDF. Accessed May 8th, 2017.
